# Risk of congestive heart failure and mortality following lymphovenous anastomosis: a nationwide population-based retrospective cohort study

**DOI:** 10.1097/JS9.0000000000000946

**Published:** 2023-11-24

**Authors:** Joon Seok Lee, Hyun Su Kang, Jae-Ho Chung, Jeong Yeop Ryu

**Affiliations:** a Department of Plastic and Reconstructive Surgery, School of Medicine, Kyungpook National University, Daegu; b Department of Plastic and Reconstructive Surgery, Korea University Hospital, Seoul, Republic of Korea

**Keywords:** heart failure, interdisciplinary research, lymphaticovenous anastomosis, lymphedema

## Abstract

**Background:**

Lymphovenous anastomosis (LVA) enables lymphatic fluid to drain into the venous system. However, no study has investigated the association between LVA and heart failure (HF) caused by fluid overload in the blood circulating system. The purpose of our study was to determine whether LVA increases the risk of HF and mortality.

**Material and methods:**

This nationwide retrospective study evaluated a total of 1400 lymphedema patients who underwent LVA and two control cohorts with 28 000 lymphedema who did not undergo LVA and 70 000 age-matched and sex-matched participants from the Korean National Health Insurance database were included. Blood pressure, body mass index (BMI), glucose and cholesterol levels, smoking history, and comorbidities were obtained during National Health Insurance Service – Health Screening (NHIS-HealS). The incidence, adjusted risk for HF, and mortality were evaluated.

**Results:**

Adjusted HRs for HF were 1.20 (confidence interval [CI], 1.03–1.40) and 1.30 (CI, 1.12–1.50), referenced by the general population control cohort and patients with lymphedema without LVA, respectively. In age, sex, BMI, and smoking status-stratified analyses, heightened risk of HF was evident across all sexes, spanning both young and old age groups, encompassing individuals with various smoking statuses, and those with a BMI of 18.5 or higher. Among these groups, the risk was notably greater in males compared to females, higher in younger individuals as opposed to older ones, and further elevated within the BMI range of 18.5–25.

**Conclusions:**

LVA is associated with an increased HF risk, independent of cardiovascular risk factors and associated comorbidities. This association is prominent in participants aged <50 years, in males, and in the normal-to-obese (BMI ≥18.5 kg/m^2^) group. Among patients with lymphedema, LVA did not significantly affect mortality.

## Introduction

Highlights

- Lymphovenous anastomosis (LVA) has an increased risk of heart failure events.
- LVA led to lower patient survival rates compared to the general population.
- Among patients with lymphedema, LVA did not significantly affect mortality.

Physiological interventions for lymphedema include lymphovenous anastomosis (LVA) and vascularized lymph node transfer (VLNT). These procedures aim to reconstruct the lymphatic drainage system, enabling lymphatic fluid to drain into the venous system^1^. Previous studies have demonstrated the efficacy of LVA in reducing limb circumference and volume, not only in the early stage but also in the advanced-stage lymphedema^2–5^. Consequently, it has recently become the preferred choice for lymphedema treatment in several hospitals^6^.

Heart failure (HF) exerts a profound influence on global health and healthcare expenditures. In the United States, Medicare reimbursements totaling ~$4.0 billion were disbursed in relation to HF in the year 2001^7^. While HF can stem from various etiologies, fluid overload stands as a significant contributory factor, resulting in an excessive accumulation of fluid within the circulatory system. This phenomenon can augment the cardiac workload due to the increased blood volume (BV) within the body. Moreover, fluid overload can precipitate HF by elevating vascular pressure, which can lead to adverse left ventricular remodeling with impaired myocardial contraction and/or relaxation, thereby impeding the heart’s ability to efficiently pump blood throughout the body. Additionally, fluid overload carries the potential to inflict damage upon the myocardium, as the surplus fluid exerts pressure upon the heart muscle, diminishing its blood supply. Over time, this cumulative damage can compromise the myocardial strength, rendering it less effective in its role of circulatory propulsion^7,8^. Furthermore, the interstitial compartment is conventionally characterized by low compliance, implying a limited capacity to accommodate fluid retention. Nevertheless, in cases of sustained impairment of lymphatic drainage, such as in chronic HF or regional lymphedema, the interstitium undergoes a transition toward a state of increased compliance, leading to a net accumulation of fluid within the interstitial space^9^. Of particular note, it has been observed that this heightened compliance state may prove to be irreversible^10^. As a result, individuals suffering from chronic lymphedema may be inherently more prone to retaining excess fluids compared to the general population without lymphedema. This heightened susceptibility to chronic fluid overload may contribute to cardiac remodeling.

An effective LVA permits the redirection of existing or newly generated lymphatic fluid into the venous system. However, transferring lymphatic fluid into the bloodstream can lead to an excess of fluid within the circulatory system. The continual infusion of lymphatic fluid into the vascular circulation system can precipitate an excess of BV. This phenomenon can give rise to pathological BV expansion, thus contributing to volume overload and subsequent organ congestion. Volume overload elicits an increase in central filling pressures, eventually culminating in symptomatic clinical congestion^7^. Furthermore, volume overload may incite pressure overload within the pulmonary vasculature, subsequently impacting cardiac function. Prolonged pressure overload on the heart can lead to left ventricular remodeling, ultimately precipitating the onset of HF^8^.

Nevertheless, there has been no research on the effects of LVA on the heart. HF can have severe consequences, including death. Therefore, this study aimed to assess the risk of HF and mortality in patients who have undergone LVA.

## Methods

### Data source

This nationwide cohort study utilized data from the Korean National Health Insurance (NHI) claims database, which provides coverage to over 97% of the entire Korean population, offering comprehensive information on medical practices in the country^11^. The NHI database contains various data points, including personal details, including birth year, sex, and region of the insured individuals, as well as socio-economic information, including income quintiles. Moreover, it encompasses exhaustive records of outpatient and inpatient claims, disease diagnoses, and treatment codes based on the International Classification of Diseases Tenth Revision (ICD-10). Additionally, the database includes results from the National Health Insurance Service – Health Screening (NHIS-HealS) program, which mandates annual or biennial health screenings for all enrolled adults in Korea. Considering the high enrollment rate of over 97%, the majority of Koreans are expected to undergo health screenings, with ~70% participation rate in health examinations^11–13^. These health screenings encompass a wide range of data, including blood and urine test results, as well as information on alcohol and smoking history.

### Study population and outcomes

**Figure 1:**
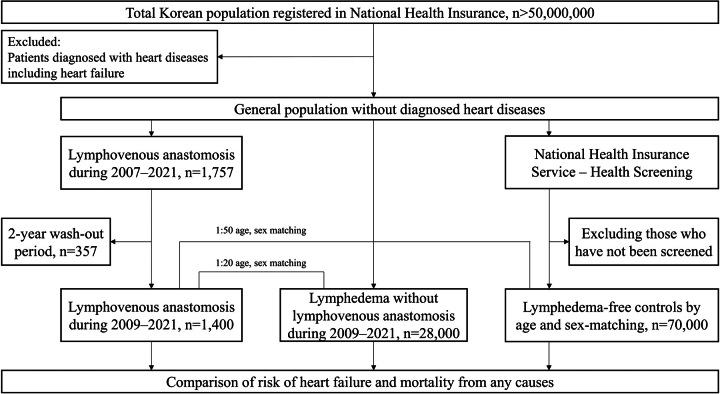
Flowchart of the study.

Patients diagnosed with lymphedema who underwent LVA between 2007 and 2021 were identified from the NHI database (Fig. 1). Populations with prior heart disease were excluded as follows: I05–09, Chronic rheumatic heart diseases; I11.0, Hypertensive heart disease with (congestive) heart failure; I13.0, Hypertensive heart and renal disease with (congestive) heart failure; I13.2, Hypertensive heart and renal disease with both (congestive) heart failure and renal failure; I20–25, Ischemic heart diseases; I26–28, Pulmonary heart disease and diseases of pulmonary circulation; I30–52, Other forms of heart disease; P29.0, Neonatal cardiac failure. Lymphedema cases were defined as those who had visited the clinic at least twice within the first year of diagnosis and were assigned one of the following ICD codes: I890 (Lymphoedema, NEC), I972 (Postmastectomy lymphoedema syndrome), or Q820 (Hereditary lymphoedema). From the cohort of patients with lymphedema, we specifically identified those who underwent LVA (P2137) and VLNT (SA16) procedures. A validation study for diagnostic accuracy was performed by reviewing the medical records of patients with lymphedema who underwent LVA at two medical centers. By calculating the sensitivity and specificity of the algorithm, these validations were performed by two plastic surgeons. Sensitivity was defined as the percentage of actual patients who met the diagnostic criteria, whereas specificity was defined as the percentage of patients without LVA who did not meet the diagnostic criteria. The sensitivity of patients with lymphedema who underwent LVA or VLNT was 83.3% and 87.5%, respectively, and the specificity was 94.74% and 91.91%, respectively.

A 2-year washout period was applied to ensure the identification of newly diagnosed patients who underwent LVA. This period spanned from January 2007 to the date of LVA surgery. The ‘washout period’ is a defined time frame during which a LVA or lymphedema is allowed to dissipate within the cohort under investigation before conducting a detailed assessment of its influence. During this period, subjects or participants are intentionally shielded from the specific variable. For the control groups, two distinct cohorts were defined. The first control group comprised patients diagnosed with lymphedema but who did not undergo LVA. For each patient who underwent LVA, we selected 20 patients with lymphedema without LVA from the NHI database, matched according to birth year, sex, and year of NHIS-HealS. The second control group included individuals without a diagnosis of lymphedema and no LVA history. For every patient who underwent LVA, we randomly selected 50 individuals from the general population who were free of both lymphedema and LVA, also matched according to birth year, sex, and year of NHIS-HealS.

The study’s primary outcomes included the occurrence of HF and mortality. HF cases were identified as both outpatients and inpatients diagnosed using the I09.9, I11.0, I13.0, I13.2, I25.5, I42.0, I42.5–I42.9, I43.x, I50.x, and P29.0 ICD-10 codes^14^. Mortality data were obtained from the NHI database, which provided comprehensive information on the death status and date of death for all Korean citizens. Additionally, we used the Charlson comorbidity index (CCI) to identify comorbidities that could impact outcomes, particularly mortality^15,16^. In addition to the standard ICD-10 code definitions in the CCI, we included heart failure-related comorbidities such as chronic kidney disease (N18.x), hyperthyroidism (E05.x), rheumatic disease (M05.x, M06.x, M31.5, M32.x–M34.x, M35.1, M35.3, M36.0), breast cancer (C50.x), and cardiomyopathy related to pregnancy (O10.1, O10.3, O29.1, O89.1, O90.3). All records 1 year prior to the date of the index hospitalization were retrieved to identify comorbidities. Comorbidities were defined in the previous admissions using major and secondary diagnoses without consideration of diagnosis type^16^. This work has been reported in line with the STROCSS, Supplemental Digital Content 2, http://links.lww.com/JS9/B401 (Strengthening The Reporting of Cohort Studies in Surgery) criteria^17^.

### Statistical analysis

This study aimed to explore the potential HF risk in patients with lymphedema who received treatment through LVA, considering several factors, including age, sex, body mass index (BMI), cardiovascular risk profiles (CVRPs) encompassing systolic and diastolic blood pressure (BP), fasting blood glucose (FBG), cholesterol levels, and smoking status. All variables were categorized and presented as frequencies and percentages. The crude incidence rate (IR) for HF was calculated as the number of HF events per 1000 person-years. We further analyzed the IR and incidence rate ratios (IRRs) according to sex, age groups (<50 and ≥50 years), and smoking status. The cumulative incidence of HF events in the three cohorts was visually represented using Kaplan–Meier curves. The follow-up period commenced on the index date and continued until the occurrence of HF events or the last day of follow-up (31 December 2021). We employed the Cox proportional hazard model and calculated hazard ratios (HRs) with a 95% confidence interval (CI) to determine the independent association between LVA and HF risk. The analysis was adjusted for sex, age groups, BMI, CVRPs, smoking status, and comorbidities that increase the risk of HF through multivariable adjustment. We further stratified the analyses by sex, age groups (<50 and ≥50 years), BMI groups, and smoking status.

Mortality data were extracted from the NHI database, providing us with the date of death for all participants, thereby allowing the calculation of person-years for the study population. We compared mortality rates among the three cohorts on the basis of the standardized mortality ratio (SMR; deaths among patients with LVA divided by deaths in the general population) and its 95% CI. Additionally, to explore the independent association between LVA and mortality, the Cox proportional hazard model was utilized, with adjusted HRs calculated and their 95% CI, accounting for sex, age group, CCI, CVRPs, BMI, and smoking status as covariates.

All statistical tests employed a two-tailed 95% CI, with a significance level set at *P*<0.05. Data analyses were performed using STATA Stata/MP2 (version 18.0; StataCorp, College Station, Texas, USA).

## Results

### Characteristics of the study population

A total of 1400 patients who met the inclusion criteria underwent LVA in this study. Among them, 119 (8.5%) and 1281 (91.5%) were male and female, respectively. A significant portion of participants, 799 (57.07%), were aged over 50 years at the time of lymphedema diagnosis.

For comparison, we identified 28 000 matching controls with lymphedema who did not undergo LVA, using a 1:20 matching ratio. Additionally, we identified 70 000 matching controls without lymphedema for the 1:50 matching ratio. The proportion of patients who underwent LVA and had a high BMI and smoking rate was lower than that of the age-matched and sex-matched general population control cohort. Conversely, similar proportions of patients who underwent LVA exhibited high BP, FBG, and total cholesterol levels compared with that of the general population control cohort. Regarding comorbidities, a distinction was observed between the lymphedema group and the control group, depending on the presence or absence of breast cancer. This observation suggests that among the factors contributing to lymphedema, a substantial number of patients in this study had secondary lymphedema associated with breast cancer. It also implies that a significant number of LVA procedures were performed on breast cancer patients. Further details regarding these characteristics are presented in Table 1.

**Table 1:** Baseline characteristics of patients with lymphovenous anastomosis and matched controls.

	Total (%)	LVA+ (%)	LVA− (%)	MC (%)
Sex				
Male	8449 (8.5)	119	2380	5950
Female	90 951 (91.5)	1281	25 620	64 050
Age (years)				
<10	284 (0.29)	4	80	200
10–20	1207 (1.21)	17	340	850
20–30	2343 (2.36)	33	660	1650
30–40	10 366 (10.43)	146	2920	7300
40–50	28 471 (28.64)	401	8020	20 050
50–60	31 240 (31.43)	440	8800	22 000
60–70	19 028 (19.14)	268	5360	13 400
70–80	5751 (5.79)	81	1620	4050
80–90	710 (0.71)	10	200	500
BMI (kg/m^2^)				
<18.5	3639 (3.66)	55 (3.93)	1012 (3.61)	2572 (3.67)
18.5–25	66 453 (66.85)	943 (67.36)	17 897 (63.92)	47 613 (68.02)
>25	29 308 (29.48)	402 (28.71)	9091 (32.47)	19 815 (28.31)
Systolic BP (mmHg)				
<120	47 283 (47.57)	699 (49.93)	13 908 (49.67)	32 676 (46.68)
120–140	44 354 (44.62)	613 (43.79)	12 117 (43.28)	31 624 (45.18)
>140	7763 (7.81)	88 (6.29)	1975 (7.05)	5700 (8.14)
Diastolic BP (mmHg)				
<80	62 269 (62.64)	952 (68.00)	17 815 (63.62)	43 502 (62.15)
80–90	32 215 (32.41)	390 (27.86)	8889 (31.75)	22 936 (32.77)
>90	4916 (4.95)	58 (4.14)	1296 (4.63)	3562 (5.09)
FBG (mg/dl)				
<100	70 786 (71.21)	1030 (73.57)	20 067 (71.67)	49 689 (70.98)
100–125	22 939 (23.08)	313 (22.36)	6296 (22.49)	16 330 (23.33)
>125	5675 (5.71)	57 (4.07)	1637 (5.85)	3981 (5.69)
Total chol (mg/dl)				
<200	52 631 (52.95)	765 (54.64)	15 140 (54.07)	36 726 (52.47)
200–240	31 268 (31.46)	441 (31.50)	8436 (30.13)	22 391 (31.99)
>240	15 501 (15.59)	194 (13.86)	4424 (15.80)	10 883 (15.55)
Smoking				
Smoker	12 714 (12.79)	199 (14.21)	3991 (14.25)	8524 (12.18)
Nonsmoker	86 686 (87.21)	1201 (85.79)	24 009 (85.75)	61 476 (87.82)
Comorbidities				
Chronic kidney disease	303 (0.30)	12 (0.85)	120 (0.43)	171 (0.24)
Hyperthyroidism	260 (0.26)	9 (0.64)	107 (0.38)	144 (0.21)
Rheumatic disease	565 (0.57)	13 (0.93)	255 (0.91)	297 (0.42)
Breast cancer	4875 (4.90)	543 (38.79)	3569 (12.75)	763 (1.09)
Cardiomyopathy-related pregnancy	0	0	0	0

BMI, body mass index; BP, blood pressure; chol, cholesterol; FBG, fasting blood glucose; LVA, lymphovenous anastomosis; MC, matched control.

### IRs and risk factors for HF events

**Figure 2:**
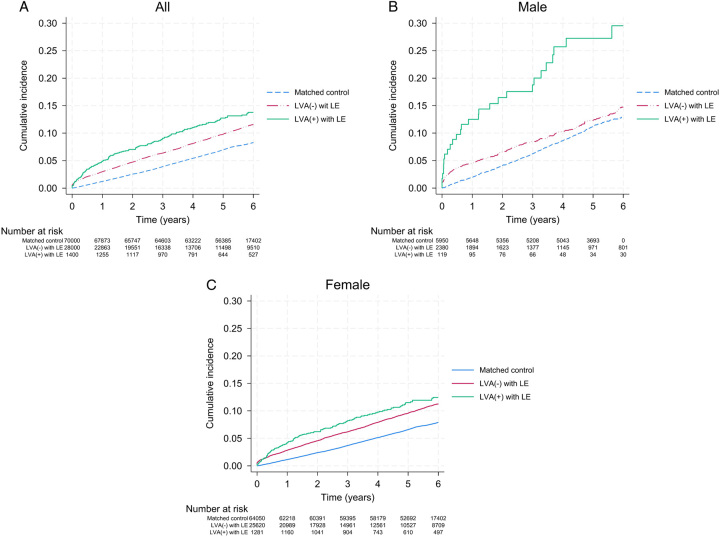
Kaplan–Meier curves for the incidence of heart failure events in patients with lymphedema who underwent lymphovenous anastomosis and control cohorts. Kaplan–Meier curves for (A) all, (B) male, and (C) female. LVA, lymphovenous anastomosis.

The cumulative incidence of HF events among the study cohorts is illustrated in Figure 2. Patients who underwent LVA exhibited a higher cumulative incidence of HF events than both non-LVA-treated lymphedema patients and matched control participants. This difference in cumulative incidence remained constant throughout the follow-up period. The specific numbers of HF events, IRs, and IRRs for HF are presented in Table 2. A total of 8187 HF events were recorded over the follow-up period. Among patients with lymphedema, 210 HF cases occurred in those who underwent LVA, whereas 2798 HF cases occurred in those without LVA. The IRs for HF were 28.61 and 21.47 per 1000 person-years, respectively. Patients who underwent LVA exhibited a significantly higher risk of HF than both control groups, with an IRR of 1.98 (CI, 1.73–2.26). Moreover, patients with lymphedema without LVA showed an increased HF risk compared with the general population, with an IRR of 1.48 (CI, 1.43–1.54). When stratified by sex and age, higher and further increased HF risks were observed in male participants and those aged <50 years.

**Table 2:** Incidence rates of heart failure events in patients with/without lymphovenous anastomosis in lymphedema and control cohorts.

	LVA (+) with LE			LVA (−) with LE			Matched control	
	Events/PYS	IR^a^ (CI)	IRR (CI)	Events/PYS	IR^a^ (CI)	IRR	Events/PYS	IR^a^ (CI)
All	210/7339.1	28.61 (24.99–32.76)	1.98 (1.73–2.26)	2798/130 300.7	21.47 (20.69–22.28)	1.48 (1.43–1.54)	5179/357 593.6	14.48 (14.09–14.88)
Male	30/499.9	60.01 (41.96–85.83)	2.55 (1.78–3.65)	293/10 873.77	26.95 (24.03–30.21)	1.15 (1.02–1.29)	646/27 477.77	23.51 (21.77–25.39)
Female	180/6839.2	26.32 (22.74–30.46)	1.92 (1.66–2.22)	2505/119 427	20.98 (20.17–21.81)	1.53 (1.47–1.59)	4533/330 115.8	13.73 (13.34–14.14)
Age <50	69/3498.13	19.72 (15.58–24.97)	2.23 (1.76–2.82)	784/64 768.62	12.10 (11.29–12.98)	1.37 (1.27–1.47)	2120/239 391.9	8.86 (8.49–9.24)
Age ≥50	141/3840.97	36.71 (31.12–43.30)	1.42 (1.20–1.67)	2014/65 532.11	30.73 (29.42–32.10)	1.19 (1.14–1.24)	3059/118 201.7	25.88 (24.98–26.81)
Nonsmoker	160/5727.01	27.94 (23.93–32.62)	2.01 (1.72–2.35)	2239/112 254.6	19.95 (19.14–20.79)	1.44 (1.38–1.50)	4378/314 975.4	13.90 (13.49–14.32)
Smoker	44/1612.09	27.29 (20.31–36.68)	1.45 (1.08–1.95)	401/18 046.11	22.22 (20.15–24.51)	1.18 (1.07–1.30)	801/42 618.15	18.79 (17.54–20.14)

a Per 1000 person-years.

CI, 95% confidence interval; IR, incidence rate; IRR, incidence rate ratio; LVA, lymphovenous anastomosis; PYS, person-years.

The adjusted HRs for HF events in relation to lymphedema with or without LVA are presented in Table 3. Even after adjusting for sex, age, various CVRPs, and associated comorbidities, LVA remained an independent risk factor for HF, with an HR of 1.20 (CI, 1.03–1.40). Furthermore, female sex, age over 70 years, BMI over 25 kg/m^2^, high BP, high FBG level, smoking, chronic kidney disease, hyperthyroidism, rheumatic disease, and breast cancer were independently associated with HF. In the hazard model comparing patients with lymphedema, those who underwent LVA had an HR of 1.30 (CI, 1.12–1.50) compared with those with lymphedema who did not undergo LVA. However, VLNT did not emerge as an independent risk factor for HF in either the general population or the lymphedema control group (Supplementary Table 1, Supplemental Digital Content 1, http://links.lww.com/JS9/B400).

**Table 3:** Adjusted risk for heart failure events of lymphovenous anastomosis by the Cox proportional hazard model.

	General population			Within lymphedema cohort		
	HR	CI	*P*	HR	CI	*P*
Patients						
** **Normal	1			N/A		
** **Lymphedema without LVA	0.96	0.91–1.02	0.153	1		
** **Lymphedema with LVA	1.20	1.03–1.40	0.016	1.30	1.12–1.50	0.001
Sex						
** **Female (vs. male)	1.15	1.06–1.26	0.001	1.06	0.91–1.23	0.457
Age (years)						
** **<10	1			1		
** **10–20	0.15	0.05–0.45	0.001	0.27	0.03–2.31	0.231
** **20–30	0.23	0.08–0.63	0.004	0.35	0.05–2.56	0.298
** **30–40	0.35	0.13–0.94	0.037	0.53	0.07–3.78	0.523
** **40–50	0.70	0.26–1.86	0.471	0.93	0.13–6.69	0.946
** **50–60	1.10	0.41–2.95	0.845	1.28	0.18–9.16	0.806
** **60–70	1.94	0.72–5.18	0.189	2.20	0.31–15.74	0.432
** **70–80	2.95	1.10–7.91	0.032	3.51	0.49–25.13	0.212
** **80–90	4.11	1.47–11.49	0.007	4.62	0.63–33.78	0.131
BMI (kg/m^2^)						
** **<18.5	1			1		
** **18.5–25	0.91	0.79–1.04	0.153	0.85	0.68–1.07	0.161
** **>25	1.15	1.00–1.32	0.042	1.07	0.85–1.35	0.568
Systolic BP (mmHg)						
** **<120	1			1		
** **120–140	1.09	1.03–1.16	0.003	1.14	1.03–1.25	0.011
** **>140	1.20	1.10–1.33	<0.001	1.27	1.07–1.50	0.005
Diastolic BP (mmHg)						
** **<80	1			1		
** **80–90	1.10	1.03–1.16	0.001	1.09	0.99–1.20	0.086
** **>90	1.12	1.00–1.25	0.047	1.08	0.89–1.31	0.419
FBG (mg/dl)						
** **<100	1			1		
** **100–125	1.07	1.02–1.13	0.008	1.08	0.99–1.18	0.083
** **>125	1.32	1.22–1.43	<0.001	1.38	1.21–1.57	<0.001
Total chol (mg/dl)						
** **<200	1			1		
** **200–240	0.98	0.93–1.03	0.352	0.93	0.86–1.02	0.116
** **>240	0.91	0.86–0.97	0.005	0.94	0.84–1.04	0.244
Smoking						
** **Smoking (vs. nonsmoking)	1.28	1.19–1.37	<0.001	1.22	1.08–1.37	0.001
Comorbidities						
Chronic kidney disease	4.78	4.06–5.63	<0.001	3.24	2.48–4.22	<0.001
Hyperthyroidism	2.84	2.20–3.67	<0.001	2.56	1.76–3.71	<0.001
Rheumatic disease	2.14	1.79–2.56	<0.001	2.03	1.59–2.60	<0.001
Breast cancer	1.18	1.06–1.30	0.002	1.15	1.02–1.29	0.020

BMI, body mass index; BP, blood pressure; chol, cholesterol; CI, 95% confidence interval; FBG, fasting blood glucose; HR, hazard ratio; LE, lymphedema; LVA, lymphovenous anastomosis; N/A, not available.

**Figure 3:**
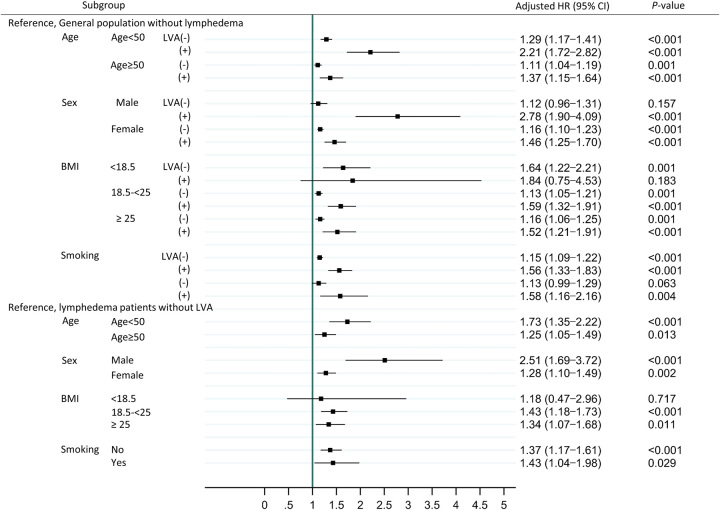
Subgroup analyses of heart failure events stratified by age, sex, body mass index, and smoking status. BMI, body mass index; CI, 95% confidence interval; HR, hazard ratio; LVA, lymphovenous anastomosis.

The subgroup analysis for HF events is presented in Figure 3. When we analyzed the lymphedema groups, a heightened risk of HF following LVA was evident across all sexes, spanning both young and old age groups, encompassing individuals with various smoking statuses and those with a BMI of 18.5 or higher. Among these groups, the risk was notably greater in males compared to females, higher in younger individuals (<50 years) as opposed to older ones, and further elevated within the BMI range of 18.5–25.

### Mortality rates between LVA and control cohorts

**Figure 4:**
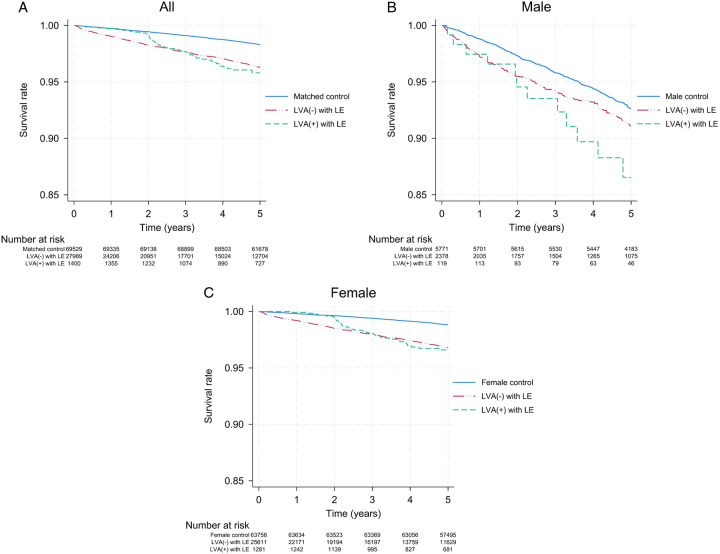
Kaplan–Meier curves for survival in patients with lymphedema who underwent lymphovenous anastomosis and control cohorts. Kaplan–Meier curves for (A) all, (B) male, and (C) female. LVA, lymphovenous anastomosis.

Data on mortality showed that of 1400 patients with lymphedema who underwent LVA, 72 experienced mortality (5.14%). Among them, the mortality rate per 1000 person-years was 8.8 for all patients, whereas it was 24.08 and 7.54 for male and female patients, respectively. Comparatively, the mortality rates per 1000 person-years for patients with lymphedema who did not undergo LVA were 7.74 for all patients, 18.86 for male patients, and 6.73 for female patients (Supplementary Table 2, Supplemental Digital Content 1, http://links.lww.com/JS9/B400). As demonstrated in Figure 4, patients with lymphedema who underwent LVA exhibited higher survival rates than those who did not undergo LVA up to 2 years from the index date, paralleling similar trends observed in non-lymphedema–matched control cohorts. However, after 2 years from the index date, survival sharply declined, with patients who underwent LVA experiencing steeper declines than those with lymphedema without LVA. The overall SMR for patients with lymphedema who underwent LVA was 2.16 (CI, 1.71–2.72). Notably, female patients showed a higher SMR than their male counterparts (2.63 vs. 1.3), and patients under the age of 50 had a higher SMR than those over the age of 50 (3.62 vs. 1.3) (Supplementary Table 3, Supplemental Digital Content 1, http://links.lww.com/JS9/B400).

Regarding adjusted HRs for mortality, the results presented in Table 4 indicate that both patients with lymphedema who underwent LVA and those who did not undergo LVA remained independent risk factors for mortality compared with the general population without lymphedema, with HRs of 1.33 (CI, 1.04–1.69) and 1.19 (CI, 1.09–1.30), respectively. However, when comparing within the lymphedema patient group, the HR of LVA was 1.09 (CI, 0.86–1.38), and it did not reach statistical significance (Table 4).

**Table 4:** Adjusted risk for mortality of patients with lymphedema with or without lymphovenous anastomosis by the Cox proportional hazard model.

	General population			Within lymphedema cohort		
	HR	CI	*P*	HR	CI	*P*
Patients						
Normal	1			N/A		
Lymphedema without LVA	1.19	1.09–1.30	<0.001	1		
Lymphedema with LVA	1.33	1.04–1.69	0.021	1.09	0.86–1.38	0.487
CCI						
(0 vs. ≥1)	3.00	2.41–3.73	<0.001	5.12	3.25–8.07	<0.001
Sex						
Female (vs. male)	0.34	0.30–0.38	<0.001	0.42	0.35–0.50	<0.001
Age						
Age <50	1					
Age ≥50	4.42	4.01–4.88	<0.001	2.76	2.39–3.18	<0.001
Systolic BP (mmHg)						
<120	1			1		
120–140	1.12	1.01–1.24	0.027	1.17	1.01–1.36	0.042
>140	1.52	1.30–1.78	<0.001	1.53	1.19–1.97	0.001
Diastolic BP (mmHg)						
<80	1			1		
80–90	0.95	0.86–1.05	0.330	0.85	0.73–0.99	0.036
>90	0.95	0.79–1.14	0.591	0.96	0.72–1.28	0.788
FBG (mg/dl)						
<100	1			1		
100–125	1.10	1.01–1.20	0.033	1.12	0.98–1.28	0.097
>125	1.68	1.49–1.89	<0.001	1.53	1.26–1.85	<0.001
Total chol (mg/dl)						
<200	1			1		
200–240	0.91	0.83–0.99	0.023	0.96	0.84–1.09	0.546
>240	0.83	0.74–0.92	0.001	0.84	0.71–1.00	0.050
BMI (kg/m^2^)						
<18.5	1			1		
18.5–25	0.54	0.45–0.65	<0.001	0.52	0.39–0.68	<0.001
>25	0.47	0.39–0.57	<0.001	0.45	0.33–0.60	<0.001
Smoking						
Smoking (vs. nonsmoking)	1.18	1.05–1.32	0.004	1.09	0.91–1.29	0.356

BP, blood pressure; CCI, Charlson comorbidity index; CI, 95% confidence interval; HR, hazard ratio; LVA, lymphovenous anastomosis; N/A, not available.

## Discussion

In this comprehensive nationwide cohort study, we meticulously identified a cohort of 1400 patients with lymphedema who underwent LVA, along with 28 000 patients with lymphedema who did not undergo LVA and an additional 70 000 matched controls. The primary objective of our investigation was to assess the potential risks of HF and mortality among patients who received LVA for their lymphedema. Remarkably, the findings from our study demonstrate a significant association between LVA and an increased risk of developing HF. Furthermore, the increased HF risk remained significant in male participants, younger age group (<50 years), and normal-to-obese participants (BMI, ≥18.5 kg/m^2^), even after adjusting for baseline CVRPs. Moreover, we observed that patients with lymphedema with or without LVA tended to have decreased survival rates compared with the general population control. Additionally, patients with lymphedema who underwent LVA also tended to have decreased survival rates compared with those who did not undergo LVA; however, this finding did not reach statistical significance. Regardless of LVA, patients with lymphedema showed statistically significantly higher mortality than age-matched and sex-matched cohorts; therefore, it can be considered that lymphedema increases the risk of mortality. However, the adjusted HR of patients with lymphedema who underwent LVA was higher than that of patients with lymphedema who did not undergo LVA. As LVA showed a statistically insignificant HR among patients with lymphedema, there is no evidence yet that LVA itself is an independent factor for mortality.

The flow of lymphatic fluid in the extremity follows the lymph vessels and enters the venous system through the thoracic duct. LVA is an attempt to introduce lymphatic fluid into the venous system earlier, before going to the thoracic duct^18^. To date, plastic surgeons have focused on the volume reduction of fluid in the extremity as the surgical effect of LVA and have failed to consider where the fluid volume reduction flows and where it ends. O’Brien *et al*. reported that among 134 patients with obstructive lymphedema, volume changes in the extremities showed a significant improvement in 22 patients (42%), with an average reduction of 44% of the excess volume. Chang *et al*. reported that the overall mean volume differential reduction of extremities was 33%, 36%, and 42% at 3, 6, and 12 months following LVA, respectively. Furthermore, Campisi *et al*. reported that volume changes in patients with lymphedema with LVA showed a significant improvement of 83%, with an average reduction of 67% of the excess volume following LVA^19–21^. Cha *et al*.^2^ even reported that the change in volume following LVA had a rapid reduction in the first week, followed by a steady decrease after 3 months. This indicates that rapid cardiac overloading occurred in the first week, followed by a steady increase after 3 months. However, all of the abovementioned studies focused on the effectiveness of reducing extremity volume in patients with lymphedema only but did not address the effects of reduced fluid volume on the cardiovascular system. Plastic surgeons who perform LVA may not experience the case of a patient with HF following LVA. However, if dyspnea, which is the main symptom of HF, develops at home following LVA, patients visit a cardiology department for a consult and do not visit plastic surgery. This is because it is difficult for patients to believe that dyspnea is caused by LVA performed during plastic surgery. If patients develop severe dyspnea, they may go to a nearby emergency department rather than to the hospital where LVA was performed. This makes it challenging for plastic surgeons to study the effects of LVA on the cardiovascular system. This nationwide population-based cohort study using the NHI database enables a comprehensive investigation of patients with lymphedema who underwent LVA at any hospital or who visited the cardiology department owing to HF. Particularly, there is a recent tendency to perform LVA not only for patients with early-stage lymphedema but also for those with advanced-stage lymphedema^2,4,22^. LVA is typically accompanied by limb compression therapy following surgery. However, evidence to date suggests that compression therapy is also contraindicated in patients with severe HF^23^. Patients with advanced-stage lymphedema may develop lymphedema for various reasons; however, it may also occur in patients with severe HF. In this case, if compression therapy is performed following LVA, the patient’s heart may deteriorate further.

As previously mentioned, LVA is a surgical technique that facilitates the redirection of stagnant lymphatic fluid into the venous system, bypassing its passage through the thoracic duct. In other words, LVA changes early-stage lymphedema from a low compliance state of interstitial fluid to a high compliance state. However, in patients with lymphedema, if the cardiovascular system has already adapted to the presence of stagnant lymphatic fluid within the circulatory system, the introduction of additional lymphatic fluid by LVA disrupts this adapted circulatory state. Consequently, it leads to a breakdown in adaptation, resulting in an increase in BV. This increased BV, in turn, imposes pressure overload on the pulmonary vessels, eventually leading to pressure overload in the heart. This, in turn, triggers left ventricular remodeling and cardiac muscular damage. Furthermore, when the heart encounters a pressure overload it cannot accommodate, it culminates in pulmonary edema, thereby exacerbating the patient’s dyspnea symptoms.

Our study had some limitations. First, because the diagnostic classification was identified using the ICD-10 code, a detailed diagnosis reflecting severity, including the clinical stage of lymphedema, was not possible. Second, data on laboratory test results, medical images, or photographs were not available. Thus, there may be a bias in the classification of diagnosis. However, the Korean NHI provides fairly accurate data. Before claiming medical fees, the ICD-10 code and surgical fees are verified by the insurance review team of each general hospital. Subsequently, Korean NHI data are stored following re-verification by the Health Insurance Review and Assessment Service, a Korean government agency^13^. Furthermore, our authors evaluated the diagnostic accuracy of classification using ICD-10 codes, which showed high sensitivity and specificity. Therefore, the risk of bias for misdiagnosis in the present study is very low. Third, we could not discriminate between LVA only and LVA with liposuction. Therefore, a group of patients with lymphedema who underwent LVA may include patients who have also undergone liposuction. Nevertheless, if the patients who underwent liposuction were mixed, the HR or IRR of our present results would be underestimated because the lymphatic fluid leaked out of the body. Fourthly, it is important to consider the potential for bias when censoring for competing events between HF and mortality. Censoring deceased patients can lead to an upward bias in the estimates of cumulative HF incidence when using the Kaplan–Meier method and the Cox model^24^. We could have explored the application of a multistate survival model to mitigate this bias. Fifthly, given that this study pertains to intervention rather than a pharmaceutical investigation, the risk of immortal time bias is comparatively lower than in pharmaceutical studies. Nevertheless, it cannot be entirely dismissed, and methodologies such as landmark analysis, which provides a potential solution, have not been employed^25^. Finally, as this study encompassed nationwide participation, it incorporated the outcomes achieved by all surgeons performing LVA procedures across the country, potentially revealing variations in the surgical proficiency among these practitioners.

This study also had several strengths. First, to the best of our knowledge, this is the first study to investigate HF risk following LVA. Second, this is the first study to investigate the mortality of patients who underwent LVA. Investigating mortality using in-hospital data cannot be free from survival bias. However, mortality studies using the NHI database are free from survival bias. Although the HR for the mortality of patients who underwent LVA among patients with lymphedema did not reach statistical significance at 1.09 (CI, 0.86–1.38), the HR for the general population control was 1.33 (CI, 1.04–1.69). Finally, among the LVA-related research reports, including recent systematic reviews, this study has the largest number of patients and demonstrates significant statistical significance. The control group was appropriately matched for age and sex, and since this study was conducted nationwide, there was minimal selection bias. Furthermore, by including medical data from the general population that cannot be obtained from hospital records, we were able to calculate not only the risk among lymphedema patients but also compare it to the general population.

## Conclusion

LVA is associated with an increased risk of HF events among patients with lymphedema, independent of conventional CVRPs and associated comorbidities. This association is more prominent in participants aged <50 years, in males, and in the normal-to-obese (BMI, ≥ 18 kg/m^2^) groups. This study shows that patients with lymphedema who underwent LVA tend to have decreased survival rates with statistical significance compared with the general population; furthermore, they have decreased survival rates compared with those who did not undergo LVA. However, this finding was not statistically significant. More careful management strategies for HF are warranted in patients with lymphedema who underwent LVA, and indiscriminate LVA in patients with lymphedema should be avoided.

## Ethical approval

This study was approved by the Institutional Review Board of Kyungpook National University Chilgok Hospital, Daegu, Korea, on 18 January 2021 (IRB No. KNUCH 2021-01-007) and performed in accordance with the principles of the Declaration of Helsinki. All personal information was anonymized.

## Consent

Written informed consent was obtained from the patient who visited our hospitals for publication and any accompanying images. A copy of the written consent is available for review by the Editor-in-Chief of this journal on request. Regarding using patients’ data in the National Health Insurance database, written informed consent was also obtained from the review committee of the National Health Insurance Sharing Service in Korea (No. NHIS-2023-1-253) for publication and any accompanying images. A copy of the written consent is also available for review by the Editor-in-Chief of this journal on request. In a study using the National Health Insurance database, it is practically impossible to obtain consent from all citizens of the country one by one, so consent is obtained from the review committee of a national institution instead of obtaining consent directly from citizens in accordance with Korean law.

## Sources of funding

None.

## Author contribution

J.Y.R. and J.S.L.: involved in the conceptualization, design, and conduct of the study; J.Y.R. and H.S.K.: planned the data curation; J.Y.R.: performed the formal analysis with help from J.C.; J.S.L. and J.C.: supplied resources for this study. All authors had access to the data. J.Y.R. and J.S.L.: performed writing – original draft of the manuscript. JYR conducted writing – review and editing. JYR is a guarantor of this work, has full access to all the data in the study, and take responsibility for the integrity of the data and the accuracy of the data analysis.

## Conflicts of interest disclosure

There are no conflicts of interest.

## Research registration unique identifying number (UIN)

Our study was registered at researchregistry.com. The unique identifying number is researchregistry9421.

## Guarantor

Jeong Yeop Ryu.

## Data availability statement

The data that support the findings of this study are available from the Korean National Health Insurance Sharing Service, but restrictions apply to the availability of these data, which were used under license for the current study, and so are not publicly available. Data are, however, available from the authors on reasonable request and with permission of the Korean National Health Insurance Sharing Service.

## Provenance and peer review

None.

## Supplementary Material
