# Supplementary material for: Risk of congestive heart failure and mortality following lymphovenous anastomosis: a nationwide population-based retrospective cohort study
Source: Int J Surg. 2023 Nov 24;110(2):1028–38. doi: 10.1097/JS9.0000000000000946 (PMC10871649; doi:10.1097/JS9.0000000000000946)
Supplement: Supplementary file 1 [file js9-110-1028-s001.docx]

**Supplemental Tables**

**sTable 1.** Adjusted risk for heart failure of vascularized lymph node transfer by the Cox proportional hazard model

**sTable 2.** Mortality rates of patients with lymphedema with/without lymphovenous anastomosis

**sTable 3.** Age- and sex-specific standard mortality ratios (SMRs) of patients with lymphedema with/without lymphovenous anastomosis

**sTable 1. Adjusted risk for heart failure of vascularized lymph node transfer by the Cox proportional hazard model**

|  |  | **General population** | | |  | **Within lymphedema cohort** | | |  |
| --- | --- | --- | --- | --- | --- | --- | --- | --- | --- |
|  |  | **HR** | **CI** | ***P*** |  | **HR** | **CI** | ***P*** |  |
| Patient | | Normal | 1 |  |  |  | N/A |  |  |
|  |  | LE without VLNT | 0.97 | 0.92–1.02 | 0.276 |  | 1 |  |  |
|  |  | LE with VLNT | 0.97 | 0.70–1.35 | 0.854 |  | 1.01 | 0.73–1.41 | 0.951 |
| Sex | Female  (vs. male) | 1.16 | 1.06–1.26 | 0.001 |  | 1.07 | 0.92–1.24 | 0.397 |  |
| Age (years) | <10 | 1 |  |  |  | 1 |  |  |  |
|  | 10–20 | 0.14 | 0.05–0.43 | 0.001 |  | 0.24 | 0.03–2.04 | 0.191 |  |
|  | 20–30 | 0.22 | 0.08–0.61 | 0.004 |  | 0.30 | 0.04–2.23 | 0.241 |  |
|  | 30–40 | 0.34 | 0.13–0.90 | 0.031 |  | 0.46 | 0.06–3.28 | 0.436 |  |
|  | 40–50 | 0.67 | 0.25–1.80 | 0.428 |  | 0.81 | 0.11–5.79 | 0.834 |  |
|  | 50–60 | 1.06 | 0.40–2.85 | 0.901 |  | 1.11 | 0.16–7.96 | 0.914 |  |
|  | 60–70 | 1.87 | 0.70–5.00 | 0.212 |  | 1.92 | 0.27–13.74 | 0.514 |  |
|  | 70–80 | 2.85 | 1.06–7.65 | 0.038 |  | 3.06 | 0.43–21.90 | 0.265 |  |
|  | 80–90 | 3.96 | 1.41–11.07 | 0.009 |  | 4.02 | 0.55–29.35 | 0.170 |  |
| BMI  (kg/m^2^) | <18.5 | 1 |  |  |  | 1 |  |  |  |
|  | 18.5–25 | 0.91 | 0.79–1.04 | 0.153 |  | 0.85 | 0.68–1.07 | 0.158 |  |
|  | >25 | 1.15 | 1.00–1.32 | 0.043 |  | 1.07 | 0.85–1.34 | 0.578 |  |
| Systolic BP  (mmHg) | <120 | 1 |  |  |  | 1 |  |  |  |
|  | 120–140 | 1.10 | 1.03–1.16 | 0.002 |  | 1.14 | 1.03–1.26 | 0.009 |  |
|  | >140 | 1.21 | 1.10–1.34 | <0.001 |  | 1.28 | 1.08–1.51 | 0.004 |  |
| Diastolic BP  (mmHg) | <80 | 1 |  |  |  | 1 |  |  |  |
|  | 80–90 | 1.10 | 1.04–1.17 | 0.001 |  | 1.08 | 0.98–1.19 | 0.108 |  |
|  | >90 | 1.12 | 1.00–1.25 | 0.050 |  | 1.08 | 0.89–1.30 | 0.441 |  |
| FBG  (mg/dL) | <100 | 1 |  |  |  | 1 |  |  |  |
|  | 100–125 | 1.07 | 1.02–1.13 | 0.008 |  | 1.08 | 0.99–1.18 | 0.081 |  |
|  | >125 | 1.32 | 1.22–1.43 | <0.001 |  | 1.37 | 1.20–1.56 | <0.001 |  |
| Total chol  (mg/dL) | <200 | 1 |  |  |  | 1 |  |  |  |
|  | 200–240 | 0.98 | 0.93–1.03 | 0.364 |  | 0.94 | 0.86–1.02 | 0.124 |  |
|  | >240 | 0.91 | 0.86–0.97 | 0.005 |  | 0.94 | 0.84–1.04 | 0.239 |  |
| Smoking | Smoking  (vs. non-smoking) | 1.28 | 1.19–1.38 | <0.001 |  | 1.23 | 1.09–1.39 | 0.001 |  |
| Comorbidities | Chronic kidney disease | 4.81 | 4.09–5.67 | <0.001 |  | 3.27 | 2.51–4.27 | <0.001 |  |
|  | Hyperthyroidism | 2.85 | 2.20–3.68 | <0.001 |  | 2.58 | 1.77–3.74 | <0.001 |  |
|  | Rheumatic disease | 2.13 | 1.78–2.55 | <0.001 |  | 2.02 | 1.58–2.59 | <0.001 |  |
|  | Breast cancer | 1.22 | 1.10–1.35 | <0.001 |  | 1.20 | 1.08–1.35 | 0.001 |  |

HR, hazard ratio; CI, 95% confidence interval; BMI, body mass index; BP, blood pressure; FBG, fasting blood glucose; chol, cholesterol; LE, lymphedema; VLNT, vascularized lymph node transfer

**sTable 2. Mortality rates of patients with lymphedema with/without lymphovenous anastomosis**

|  | **LVA (+)** | | |  | **LVA (−) with LE** | | |  | **Matched control** | | |
| --- | --- | --- | --- | --- | --- | --- | --- | --- | --- | --- | --- |
|  | **Person-years** | **Death** | **Mortality rates (CI)** |  | **Person-years** | **Death** | **Mortality rates (CI)** |  | **Person-years** | **Death** | **Mortality rates (CI)** |
| All | 8,183.8713 | 72 | 8.8 (6.98–11.08) |  | 142,186.33 | 1,101 | 7.74 (7.30–8.21) |  | 37,9041.2 | 1,545 | 4.08 (3.88–4.28) |
| Male | 622.84736 | 15 | 24.08 (14.52–39.95) |  | 11,931.554 | 225 | 18.86 (16.55–21.49) |  | 29,206.111 | 542 | 18.56 (17.06–20.19) |
| Female | 7,561.024 | 57 | 7.54 (5.82–9.77) |  | 130,254.77 | 876 | 6.73 (6.29–7.19) |  | 349,835.09 | 1,003 | 2.87 (2.70–3.05) |
| Age < 50 | 3,735.4031 | 18 | 4.82 (3.04–7.65) |  | 67,589.254 | 244 | 3.61 (3.18–4.09) |  | 248,557.34 | 331 | 1.33 (1.20–1.48) |
| Age ≥ 50 | 4,448.4682 | 54 | 12.14 (9.30–15.85) |  | 74,597.073 | 857 | 11.49 (10.74–12.28) |  | 130,483.86 | 1,214 | 9.3 (8.79–9.84) |

LVA, lymphovenous anastomosis; LE, lymphedema; CI, 95% confidence interval

**sTable 3. Age- and sex-specific standard mortality ratios (SMRs) of patients with lymphedema with/without lymphovenous anastomosis**

|  | **LVA (+)** | | |  | **LVA (−) with LE** | | |
| --- | --- | --- | --- | --- | --- | --- | --- |
|  | **Obs** | **Exp** | **SMR (CI)** |  | **Obs** | **Exp** | **SMR (CI)** |
| All | 72 | 33.36 | 2.16 (1.71–2.72) |  | 1,101 | 579.56 | 1.9 (1.79–2.02) |
| Male | 15 | 11.56 | 1.3 (0.78–2.15) |  | 225 | 221.42 | 1.02 (0.89–1.16) |
| Female | 57 | 21.68 | 2.63 (2.03–3.41) |  | 876 | 373.45 | 2.35 (2.20–2.51) |
| Age < 50 | 18 | 4.97 | 3.62 (2.28–5.74) |  | 244 | 90.01 | 2.71 (2.39–3.07) |
| Age ≥ 50 | 54 | 41.39 | 1.3 (1.00–1.70) |  | 857 | 694.04 | 1.23 (1.15–1.32) |

LVA, lymphovenous anastomosis; LE, lymphedema; Obs, observed; Exp, expected; SMR, standard mortality ratio; CI, 95% confidence interval
